# Development and validation of an assay for measurement of leptin in pig saliva

**DOI:** 10.1186/s12917-016-0871-9

**Published:** 2016-10-28

**Authors:** Elizabeth M. S. Schmidt, Damián Escribano, Silvia Martinez-Subiela, Silvia Martinez-Miró, Fuensanta Hernández, Asta Tvarijonaviciute, José J. Cerón, Fernando Tecles

**Affiliations:** 1 Department of Veterinary Clinical Sciences, School of Veterinary Medicine and Animal Science, São Paulo State University (FMVZ - UNESP), campus of Botucatu. Rua Dr. Walter Maurício Correa, s/n, Botucatu, SP 18618-681 Brazil; 2 Interdisciplinary Laboratory of Clinical Analysis (Interlab-UMU), Veterinary Clinical Hospital, Campus of Excellence Mare Nostrum, University of Murcia, Espinardo-Murcia, 30100 Spain; 3 Animal Production Department, Veterinary School, Campus of Excellence Mare Nostrum, University of Murcia, Espinardo-Murcia, 30100 Spain; 4 Department of Animal Medicine and Surgery, Veterinary School, University Autonoma of Barcelona, Bellaterra, Barcelona, 08193 Spain

**Keywords:** Inflammation, Leptin, Porcine saliva, Stress, Validation

## Abstract

**Background:**

Leptin has been measured in human in saliva samples. However, the low leptin concentration found in this biological fluid makes necessary the use of high sensitive methods. To the authors’ knowledge, leptin has not been measured in porcine saliva. This study aimed to develop and validate a time-resolved immunofluorometric assay (TR-IFMA) for salivary leptin measurements in pigs, using a species-specific antibody, and to evaluate how salivary leptin changes with body weight, food ingestion, and in experimental models of stress and inflammation. Polyclonal antibodies were produced in rabbits immunized with recombinant porcine leptin and used to develop a sandwich TR-IFMA.

**Results:**

The method had intra-assay and inter-assay coefficients of variation lower than 10 and 16 %, respectively. The assay was accurate and the low limit of detection allowed detection of leptin in all analyzed samples. Salivary leptin concentration was positively correlated to body weight (*r* = 0.58, *P* = 0.01) and increased after food ingestion (*P* < 0.001) and after 24 h of applying a model of experimental inflammation by turpentine injection (*P* < 0.05). However, it did not significantly change after a model of acute stress consisting of a nose snare restraining.

**Conclusion:**

These results indicate that the developed assay can measure leptin in porcine saliva in a reliable way and that leptin in saliva is influenced by body weight, food ingestion and inflammation.

## Background

Leptin is a circulating polypeptide of 146 amino acids [1] that is mainly produced and secreted by the white adipose tissue [2, 3]. In pigs, serum leptin increases with body weight, whereas fasting results in decreased serum leptin secretion promoting physiological adaptations to starvation [4, 5].

There are evidences of a link between leptin and mechanisms involved in stress and inflammation. Cortisol concentration is inversely related to those of leptin [1, 6] and leptin treatment inhibits cortisol synthesis by the adrenal cortex [7]. On the topic of inflammation, experimental acute endotoxemia in pigs showed two opposite mechanisms regarding leptin expression: inflammatory mediators stimulated leptin expression but the induced changes in energy metabolism reduced expression of leptin [7–9].

Saliva has various advantages compared to blood sampling. It is non-invasive, allows repeated sampling over short time intervals and can be carried out by individuals with limited training [10, 11]. In a recent study performed in pigs [12], changes on the expression of leptin receptor and the local production in the mandibular glands according to the diet effect have been reported. Therefore, as well as the leptin has been identified [13] and determined [14] in human saliva; we hypothesized that leptin could be also measured in porcine saliva. The aim of the present study was to develop and validate a time-resolved immunofluorometric assay (TR-IFMA), by using species-specific polyclonal antibodies, for the determination of leptin in the saliva of pigs, and also to evaluate the effect of body weight, food intake, and experimental models of inflammation and stress in salivary leptin concentrations.

## Methods

### Animals and sampling procedures

Saliva samples were obtained from pigs that belonged to the experimental farm unit of the University of Murcia, Spain. All animals were subjected to a clinical examination prior, and throughout, the study and no clinical signs of disease were detected. All samples were collected using saliva collection tubes (Sarstedt, Aktiengesellschaft & Co., Numbrecht, Germany) and sponges, as reported before [15]. Each pig was allowed to gently chew on a sponge, which was clipped to a flexible thin metal rod, until the sponge was thoroughly moistened (aprox 1 min). Saliva samples were collected and centrifuged for 10 min at 3000 g to collect the saliva and stored at −80 °C until analysis.

### Production of polyclonal antibody

The recombinant porcine leptin protein [16] was selected as immunogen to produce polyclonal antibodies. The specific polyclonal antibodies against porcine leptin were produced in our laboratory according to standard protocols (University of California, Berkeley Animal Care and Use Program). In brief, a 3-month-old New Zealand rabbit was immunized using 200 μg of the recombinant porcine leptin (as antigen). The blood was extracted and its immunoglobulin G (IgG) content was purified using a HiTrapTM Protein G HP column, according to the manufacturer’s instructions (GE Healthcare Life Sciences, Munich, Germany). The purity of the IgG was assessed by 4 to 12 % SDS-PAGE and quantified using RC/DC protein assay (BioRad Laboratories, Madrid, Spain).

### Antibody labeling

An aliquot of 1 mg of the produced polyclonal antibody (rabbit anti-recombinant leptin) was used as a capture antibody in the immunoassay and was labeled with biotin using a commercial kit (EZ-Link Sulfo-NHS-biotin, Pierce, Thermo Scientific, Barrington, IL, USA). An additional aliquot of 1 mg of the same polyclonal antibody was used as a detection antibody (anti-recombinant leptin) and was labeled with a europium (Eu) chelate (DELFIA Eu-labeling kit, PerkinElmer Life and Analytical Sciences, Turku, Finland), following the manufacturer’s instructions.

### Immunoassay development

Streptavidin microtitration strips (PerkinElmer Life and Analytical Sciences) were coated with 200 μL of biotinylated antibody (250 ng/well) and were incubated for 1 h at room temperature with continuous shaking. Then the strips were washed four times with 200 μL of wash buffer (PerkinElmer Life and Analytical Sciences), and 200 μL of diluted samples (1:2) or standard were added. Plates were incubated for 1 h, and after a second wash cycle, 200 μL of the Eu-labelled antibody (300 ng/well) were added to each well. The strips were incubated for 1 h and then washed again. Following, 200 μL/well of enhancement solution (PerkinElmer Life and Analytical Sciences) were added, and strips were shaken for 5 min. The enhanced fluorescence, proportional to the quantity of leptin in the sample, was measured in a VICTOR2 1420 multilabel counter (PerkinElmer Life and Analytical Sciences), and concentrations were calculated by the Wallac MultiCalc program (PerkinElmer Life and Analytical Sciences).

### Western blotting

Standard SDS-PAGE and Western blot techniques were used to document that the polyclonal antibody recognized the recombinant porcine leptin and leptin in saliva. In brief, the recombinant protein, two non-concentrated and one 10-fold concentrated saliva samples from pigs were electrophoresed in 12 % gels in the presence of dithiothreitol, and transferred to nitrocellulose membranes (GE Health Care UK Limited, Buckinghamshire, UK). After the transfer, blots were blocked with TBS-milk 5 % overnight at 4 °C, then washed and incubated with rabbit leptin polyclonal antibody for 2 h at room temperature. Additionally, the blots were incubated with anti-rabbit IgG (Goat Anti-Rabbit IgG (H + L)-HRP Conjugate #1706515, Bio-Rad Laboratories Inc, Hercules, CA, USA) for 1 h at room temperature, revealed with fluorescein (Pierce ECL Plus Western Blotting Substrate, Thermo Scientific, Rockford, IL, USA) for 5 min, and scanned for chemiluminescence in a Typhoon 9410 (GE Health Care UK Limited, Buckinghamshire, UK).

### Analytical validation

The intra-assay precision, expressed as the coefficient of variation (CV), was calculated by measuring two pools of saliva samples, containing high and low levels of leptin, respectively, six times in a single analytical run. These samples were obtained from 6 animals with similar leptin concentrations, and the leptin content was measured using the same assay described above. The same pools were used to determine the inter-assay precision by analyzing them on 4 days within a 7 day period. The samples were frozen in aliquots, and vials were only thawed as required for each analytical run in order to prevent any possible variation as a result of repeated freeze-thaw cycles. The detection limit was defined as the lowest concentration of leptin that could be distinguished from a specimen of zero value. It was calculated for the immunoassay on the basis of date from 10 replicate determinations of the zero standard (assay buffer) as mean value plus two standard deviations. As no reference assay is available to quantify porcine salivary leptin, the accuracy was indirectly investigated by three methods: linearity under dilution, recovery experiment and comparing the results obtained with TR-IFMA and a commercially available method based on radioimmunoassay (RIA, Multi-species Leptin Assay Kit, Linco Research, St. Louis, MO, USA) previously validated for porcine plasma [17]. Linearity under dilution was determined by using two porcine saliva samples with high concentrations of leptin serially diluted (1:2, 1:4, 1:8, 1:16, 1:32, 1:64) with assay buffer, and the leptin concentration was measured by the present TR-IFMA. Afterwards, curves representing salivary leptin concentration measured versus salivary leptin concentration expected were constructed, and the coefficient of correlation (r) was calculated. The recovery experiment was performed as previously reported [18], by spiking a high leptin concentration saliva sample with a low leptin concentration sample at different rates. The percentages of recovery were calculated according with the following formula: (observed results-unspiked results)/spike amount × 100. For comparing the results obtained with TR-IFMA and RIA assays, a total of 57 porcine saliva samples corresponding to the fasting experiment were analyzed by using both methods, although this model comprised 60 samples, three samples could not be used for not having enough volume for the assays.

### Use of salivary leptin as a biomarker

In order to assess the utility of salivary leptin as a biomarker in pigs, the effect of four different variables (psychological stress, inflammation, fasting and bodyweight) on salivary leptin measured by the developed TR-IFMA method was studied. The stress induction was performed by restraining the pigs with a nose-snare, a model that has shown increases in salivary cortisol [19], chromogranin A [20], and testosterone of pigs [21]. A total of 7 pigs [Duroc × (Landrace × Large White)], 60 days of age, were used. Animals were housed in groups of seven and had access to nutritionally balanced commercial diet with water ad libitum (from nipple drinkers). Each pen had slatted-floors, and an area of 1.1 m2 per animal. The selected animals were immobilized for 1 min with a nose-snare as a stress stimulus, and saliva samples were collected before (pre-stress or baseline), immediately after (T0), 15 (T15min), and 30 (T30min) minutes after (post-stress), respectively. The animals set for stress induction were from different pens in order to avoid the possible psychological stress that the animal could suffer due to the observation of nasal snare application to others. In addition to leptin, salivary concentrations of CgA were also analysed with a previously validated assay [20], in order to evaluate if an acute stress was produced in our experimental model and to evaluate its correlation with leptin.

Banked saliva samples from an experimentally induced inflammation that was performed in 5 pigs [Duroc × (Landrace × Large White], 60 days of age, by turpentine oil subcutaneous injection [22] were used in this approach. Samples were from before (pre-inoculation value; T0), 24 h (T24h), 48 h (T48h), 72 h (T72h), and 7 days (T7d) after the administration of the turpentine. Leptin was measured in the saliva samples as previously described. Serum concentrations of C-reactive protein (CRP), previously measured in this experimental model as control biomarker of inflammation, were used to study a possible correlation to the concentrations of salivary leptin.

A group of 20 female pigs (Landrace x Landrace, 28 days of first gestation) was used to study the effect of a 24 h period of fasting. All animals were housed in individual pens of 1.57 m^2^ per animal and were fasted during 24 h. After fasting they were given 2.5 kg of a commercial pregnant diet only once per day at 07:00 in the morning. They had access to water continuously available. Saliva samples were obtained at different times. The first sample was taken 15 min after 24 h of fasting and before feeding the animals (T1) and the remaining samples were taken at 0 (immediately) and 90 min after the food administration (T2 and T3, respectively).

Six male pigs (Large White × Large White) were sampled and weighted in three different time points during growing period at 60, 90, and 150 days of age. Animals were housed in groups of seven, with 1.1 m^2^ per animal, and had access to nutritionally balanced commercial diet with water ad libitum during the sampling period. Leptin was measured as previously described by using the TR-IFMA method.

### Statistical analysis

Data analyses were performed using a statistics package (GraphPad Prism 6, GraphPad Software Inc., La Jolla, CA). The salivary leptin concentrations were evaluated for normality of distribution, using Shapiro-Wilk/Kolmogorov-Smirnov tests, giving a non-parametric distribution. Spearman correlation coefficient and Bland-Altman plot (Difference vs Average) were calculated for comparison between TR-IFMA and RIA analyses. The effects of restraint, inflammation, fasting and growing on leptin concentrations were tested on a set of data containing observations from different time points using Friedman, and Dunn’s multiple comparison tests. Spearman correlations were calculated for salivary leptin and CgA concentrations of the stress model, for salivary leptin and serum CRP levels in the inflammation model and for salivary leptin and body weights in the growing model. A value of *P* < 0.05 was used to indicate significance in all analyses.

## Results

### Production of polyclonal antibody and western blotting

**Fig. 1:**
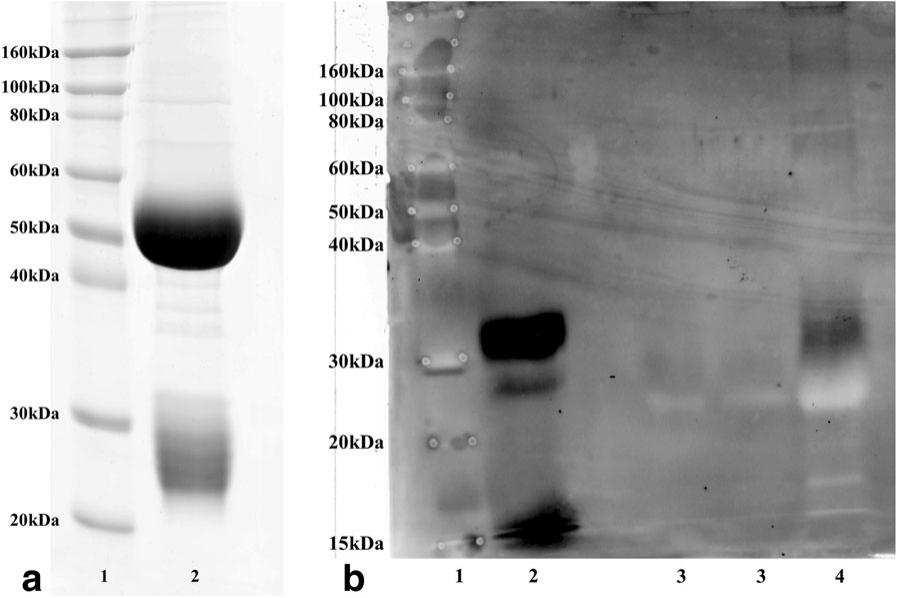
**a** SDS-PAGE of the rabbit polyclonal antibody against the recombinant porcine leptin. Lane 1: molecular weight markers (Invitrogen, Barcelona, Spain). Lane 2: purified immunoglobulin G (5 μg). **b** Western blotting of salivary leptin. Lane 1: molecular mass markers in kDa (Invitrogen, Barcelona, Spain). Lane 2: recombinant pig leptin. Lanes 3: unconcentrated pig saliva samples. Lane 4: 10-fold concentrated pig saliva sample

SDS–PAGE gel analysis of the purified IgG revealed two bands at 50 and 25 kDa, respectively, corresponding to the heavy and light IgG chains (Fig. 1a), and indicating a very high degree of purity of the polyclonal antibody. Western blotting results showed high affinity for the recombinant porcine leptin at 15 and 30 kDa roughly, and for an equivalent band in the concentrated porcine saliva at 30 kDa (Fig. 1b).

### Analytical validation

**Fig. 2:**
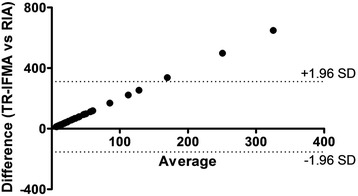
Comparisons between TR-IFMA and RIA. Bland-Altman of correlation (Difference versus average)

For calibration curve, seven concentrations were chosen for routine use: 0.39, 0.78, 1.56, 3.12, 6.25, 12.5 and 25 ng/well. The calibration curve in this range of concentration was completely linear with *r* = 0.99. All saliva samples with a degree of dilution 1:2 were within this measurement range. Results from the precision study are shown in Table 1. The intra-assay variation showed CVs lower than 10 %, whereas the inter-assay variation provided CVs lower than 17 %. The analytical limit of detection was 1.047 ng/mL. The dilution of two pig saliva samples with high leptin concentrations resulted in linear regression equations with *r* = 0.99. In the recovery experiment, the recovery average was 82.52 % (*r* = 0.98). A moderate positive correlation between TR-IFMA and RIA was observed (Spearman correlation coefficient 0.498, *P* < 0.001). Bland-Altman showed 78.5 bias with 95 % limits of agreement between −153.7 and 310.6 ng/mL (Fig. 2).

**Table 1:** Intra-assay and inter-assay obtained in saliva pools of pigs with high and low concentrations of leptin

Saliva pools (ng/mL)	Intra-assay			Inter-assay		
	Mean	SD	CV (%)	Mean	SD	CV (%)
Low (*n* = 6)	3.35	0.32	9.53	3.05	0.51	16.86
High (*n* = 6)	255.3	25.17	9.86	211.86	26.88	12.68

*SD* standard deviation, *CV* coefficient of variation

### Use of salivary leptin as a biomarker

Results from salivary leptin in all models studied are shown in Table 2.

**Table 2:** Salivary levels of leptin (median, 25th and 75th percentiles; ng/mL) in pigs submitted to different models. A stress model, where samples were taken to 7 pigs before (baseline) and immediately (T0), 15 min (T15) and 30 min (T30) after immobilization with nose-snare. A inflammation model, were samples were taken on 5 pigs before (baseline) and after turpentine injection at 24 h (T24h), 48 h (T48h), 72 h (T72) and 7 days (T7d). A fasting model, where samples were taken to 20 pigs 15 min after of 24 h of fasting and before feeding the animals (T1), immediately (T2) and 90 min (T3) after the food administration. Finally, a growing model where samples were taken to 6 pigs at 60, 90, and 150 days of age

Stress model		Inflammation model		Fasting model		Growing model	
*n* = 7		*n* = 5		*n* = 20		*n* = 6	
Time of sampling	Median (25–75^th^)	Time of sampling	Median (25–75^th^)	Time of sampling	Median (25–75^th^)	Time of sampling	Median (25–75^th^)
Baseline	32.1 (16–90)	Baseline	28.6 (24–36)	T1	25.7† (21–36)	60 days	21.9 (19–37)
T0	19.1 (13–72)	T24h	72.3* (55–156)	T2	117.8* (79–255)	90 days	36.1 (27–83)
T15	16.6 (12–29)	T48h	36.0 (30–90)	T3	24.1† (16–41)	150 days	42.8* (36–84)
T30	13.6 (8–56)	T72h	40.3 (37–151)				
		T7d	31.6† (24–33)				

* Significant (*P* < 0.05) difference with the first sampling (Baseline, T1 and 60 days, for inflammation, fasting and growing models respectively)

† Significant (*P* < 0.05) difference compared to second sampling (T24h for inflammation model and T2 for fasting model)

There were no significant differences for salivary leptin concentrations between baselines, immediately after, 15 min, or 30 min after the pigs were subjected to the nose-snare stressor stimulus. However, salivary CgA concentration was significantly increased (*P* < 0.01) in pigs 15 min after the stressor stimulus with a median value of 1.49 μg/mL, compared with the basal sample that had a value of 0.84 μg/mL. Salivary leptin and CgA were poorly correlated (*r* = 0.15, *P* = 0.127). In experimental inflammation, salivary leptin concentration showed a significant increase (*P* < 0.05) after 24 h of turpentine injection. In addition, a significant decrease in salivary leptin concentration (*P* < 0.05) was observed between T24h and T7d. Salivary leptin and serum CRP showed a slight positive correlation in this model (*r* = 0.398, *P* = 0.049).

In fasting animals, a significant increase (*P* < 0.001) in leptin was observed at T2 compared to values obtained just after the fasting period (T1), returning to baseline values at T3. When the relation between bodyweight and leptin was studied, the mean (±SD) body weights at 60, 90, and 150 days of age were 21.2 ± 2.7; 38.5 ± 3.9; and 92 ± 6 kg, respectively and salivary leptin concentrations were significantly increased (*P* = 0.0117) at 150 days of age compared to 60 days of age. Salivary leptin and body weights were positively correlated (*r* = 0.58, *P* = 0.01).

## Discussion

In this study, a TR-IFMA for the measurement of salivary leptin concentration in pigs was developed and validated. The CVs obtained for intra-assay precision were below 10 %, whereas the inter-assay CVs were below 17 %, in all cases, and it is generally accepted that the CVs must be lower than 20 % for immunological assays [23]. The assay showed high correlation coefficients in serially diluted samples of saliva and high recovery rates of leptin. Furthermore, the assay correlated with a RIA assay, showing a proportional error but having the advantage comparing with the RIA that did not use radioactive material and which did not need previous lyophilization as the RIA did in some samples with low leptin concentration. The overall results of the analytical validation that were assessed indicated that the method was adequate for detecting leptin in pig saliva.

In our study, no changes in leptin concentrations were observed in the nose-snare model of stress. This would indicate that leptin is not affected by this stress model and possible acute stress does not influence leptin concentrations in saliva. However a significant increase in salivary leptin concentrations was observed with the experimental model of inflammation of our study. In addition, a significant correlation was observed when compared to an inflammatory biomarker such as CRP. There are evidences of the role of leptin as an immunomodulator as leptin receptors are expressed in peripheral blood mononuclear cells, of the hematopoietic and immune systems, mediating its proliferation and activation. In addition cytokines such as interleukin-1 and 6 and tumor necrosis factor-alpha can act on adipocytes inducing leptin secretion in sick/diseased mammals such as pigs [4, 7, 24]. These data support the hypothesis regarding leptin as a proinflammatory cytokine with a possible role as a link between the nutritional status and the immune response [3, 25].

Since the inflammatory response induced by turpentine in our experiment did not affect food intake (data not shown), fasting would not influence the leptin concentrations in our model contrarily to results from other inflammatory models where fasting produced a leptin decrease [8].

Salivary leptin increased with body weight in our study, being both parameters correlated. Leptin is mainly produced in the white adipose tissue and the expression and secretion of this protein is significantly correlated to adipocyte size and body fat mass as it has been described in rodents and humans [1, 2]. In pigs, a high leptin concentration in serum has been related to increased body weight, since the expression of leptin mRNA was described to be weight dependent [4, 7, 26].

Salivary leptin increased when pigs were re-fed after food restriction. Leptin is an anorexigenic agent which regulates food intake [27]. In addition, its effects on intake are centrally mediated in a fast way [28], which could explain the rapid increase found in our study. Therefore, salivary leptin concentration could be a biomarker of the nutritional status as it has been observed with serum leptin in various species [4, 7].

It is important to note that different number of animals was used in the different experiments and this could be considered as a limitation of the study. This was due to ethical reasons, therefore low number of animals was included for the inflammatory and stress experiments, whereas for the fasting experiment a higher sample population could be used.

## Conclusions

A novel immunofluorometric assay for measuring salivary leptin concentrations in pigs was developed, showing good precision, sensitivity and accuracy. In addition, salivary leptin concentrations in pigs positively correlated to body weight and increased with refeeding after a fasting period and after an inflammatory stimulus.

## Abbreviations

CgA: Chromogranin A
CRP: C-reactive protein
CV: Coefficient of variation
Eu: Europium
IgG: Immunoglobulin G
RIA: Radioimmunoassay
SDS-PAGE: Sodium dodecyl sulphate polyacrilamide gel electrophoresis
TR-IFMA: Time resolved immunofluorometric assay
